# Primary Adrenal Lymphomas with Cushing’s Syndrome: Two Cases with Evidence of Endogeneous Cortisol Production by the Neoplastic Lymphoid Cells

**DOI:** 10.3390/jcm12155032

**Published:** 2023-07-31

**Authors:** Sotirios G. Papageorgiou, Ioanna Mavroeidi, Marios Kostakis, Aris Spathis, Danai Leventakou, Evangelia Kritikou, Nikolaos Oikonomopoulos, Chrysoula Kourkouti, Maria Krania, Anthi Bouchla, Thomas Thomopoulos, Zoi Tsakiraki, Konstantinos Markakis, Ioannis G. Panayiotides, Nikolaos Thomaidis, Vasiliki Pappa, Periklis G. Foukas, Melpomeni Peppa

**Affiliations:** 1 Hematology Unit, Second Propaedeutic Department of Internal Medicine and Research Institute, University General Hospital “Attikon”, School of Medicine, National and Kapodistrian University of Athens, 12462 Athens, Greece; anthibouhla@hotmail.com (A.B.); th.thomopoulos@gmail.com (T.T.); vas_pappa@yahoo.com (V.P.); 2 Endocrine Unit, Second Propaedeutic Department of Internal Medicine and Research Institute, University General Hospital “Attikon”, School of Medicine, National and Kapodistrian University of Athens, 12462 Athens, Greece; joannamavroeidi@gmail.com (I.M.); chrysoula.kourkouti@gmail.com (C.K.); maroulionline@yahoo.gr (M.K.); moly6592@yahoo.com (M.P.); 3 Laboratory of Analytical Chemistry, Department of Chemistry, National and Kapodistrian University of Athens, 15772 Athens, Greece; makostak@chem.uoa.gr (M.K.); evkritik@chem.uoa.gr (E.K.); ntho@chem.uoa.gr (N.T.); 4 Second Department of Pathology, University General Hospital “Attikon”, School of Medicine, National and Kapodistrian University of Athens, 12462 Athens, Greece; arisspa@gmail.com (A.S.); danaileventakou@gmail.com (D.L.); zoi_tsa@hotmail.com (Z.T.); ioagpan@med.uoa.gr (I.G.P.); 5 Second Department of Radiology, University General Hospital “Attikon”, School of Medicine, National and Kapodistrian University of Athens, 12462 Athens, Greece; nick.oikonomopoulos@gmail.com; 6 Second Propaedeutic Department of Internal Medicine and Research Institute, University General Hospital “Attikon”, School of Medicine, National and Kapodistrian University of Athens, 12462 Athens, Greece; kmarkakis@med.uoa.gr

**Keywords:** primary adrenal lymphoma, non-Hodgkin lymphoma, diffuse large B-cell lymphoma, autonomous cortisol secretion, adrenal incidentaloma, Cushing’s syndrome

## Abstract

Primary adrenal lymphoma (PAL) is a rare entity that presents as unilateral or bilateral rapidly growing adrenal masses, with signs and symptoms most commonly related to adrenal insufficiency due to the mass effect on the surrounding tissues. Although steroeidogenesis has not been previously described in PAL, we herein report two cases of PAL presenting as adrenal incidentalomas (AIs) that demonstrated autonomous cortisol production. A 52-year-old woman presented with lumbar pain; a computed tomography (CT) scan demonstrated a left AI measuring 8.5 × 15 × 10 cm. Similarly, an 80-year-old woman presented with lumbar pain, demonstrating in a CT scan a bilateral AI (right: 9 × 6.5 cm, left: 3.6 × 3.2 cm). Both cases underwent a full hormonal evaluation according to the algorithm for the investigation of AIs, demonstrating increased 24-h cortisol excretion, suppressed fasting adrenocorticotropic hormone (ACTH) levels, and non-suppressed serum cortisol levels in both the overnight and the low-dose dexamethasone suppression tests, indicating autonomous cortisol secretion and Cushing’s syndrome. In a relatively short time, both patients developed night sweats, and their clinical picture deteriorated, while the CT scans showed increased dimensions of the masses with radiological characteristics compatible to lymphoma. Both patients underwent ultrasound-guided biopsies (FNBs), revealing infiltration of the left adrenal by diffuse large B-cell lymphoma in the first case, whereas bilateral adrenal infiltration from the same histological type was noted in the second case. Subsequently, they were treated with immunochemotherapy, but the second patient died from an infection shortly after the initiation of the treatment. To our knowledge, this is the first report of PAL presenting with Cushing’s syndrome due to autonomous cortisol production, indicating that neoplastic lymphoid cells in PAL might acquire the potential for steroidogenesis; therefore, more cases of PAL should be analyzed so as to further elucidate the complex pathogenesis and the natural course of this entity.

## 1. Introduction

Primary adrenal lymphoma (PAL) is considered a rare pathological entity, accounting for less than 1% of all non-Hodgkin lymphoma (NHL) cases, and most commonly presenting as bilateral adrenal masses without any other extra-adrenal involvement [1,2].

PAL primarily affects older males and presents with a wide spectrum of symptoms. A minority of patients might remain asymptomatic until late in the disease course; notably, in several cases, the lymphomatous involvement of the adrenals has been discovered upon postmortem examination [3]. More commonly, upon progression, the rapidly growing adrenal masses exert mass effects on the neighboring organs and tissues, producing related signs and symptoms such as lumbar pain and adrenal insufficiency, as well as non-specific symptoms such as fatigue, weakness, and nausea related to the neoplastic nature of the disease [4,5]. Almost all PAL cases are of B-cell origin and, in the vast majority of cases, histopathology reveals diffuse large B-cell lymphoma (DLBCL) [5].

Despite its rarity, PAL should be considered in the differential diagnosis of rapidly growing unilateral or bilateral adrenal incidentalomas (AI) that demonstrate high prevalence due to the increased utility of abdominal computed tomography (CT) scans [6,7]. The majority of AIs are benign, unilateral or bilateral, functioning (25%) or non-functioning (75%) adenomas. Less than 5% are malignant, 80% of them representing adrenocortical cancer and the rest metastases from lung, breast, kidney, and colon cancers, as well as melanoma and lymphoma [8]. In this context, the latest guidelines for the management of AI recommend a specific work-up algorithm in order to assess whether the AI is benign or malignant and to exclude excessive cortisol production [8,9].

We herein report two cases of AI, proven to be PAL with autonomous cortisol secretion Cushing’s syndrome.

## 2. Case Presentation

### 2.1. Case 1

**Figure 1:**
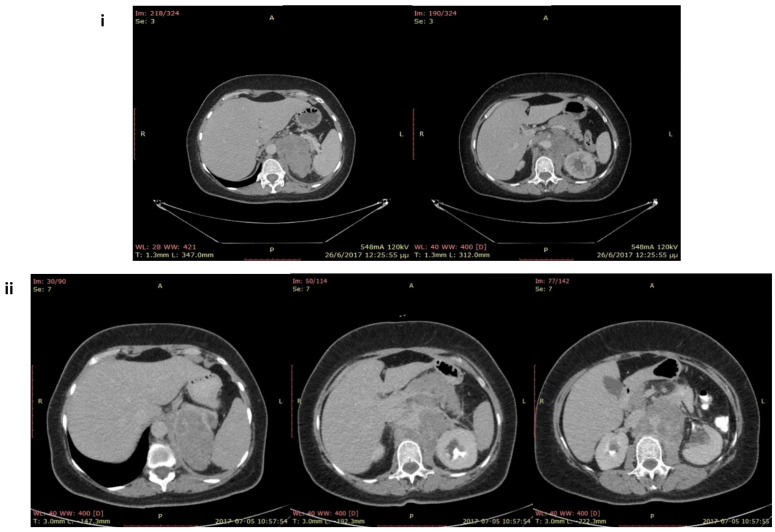
CT scans of the upper and lower abdomen from the first patient: (**i**) first CT scan (at the initial diagnosis with a max diameter of 8.5 × 7 × 4.2 cm; (**ii**) second CT scan without contrast in sequential incisions revealed a large left adrenal mass with a max diameter of 8.5 × 10 × 15 cm after 20 days.

A 51-year-old female presented with lumbar pain that had worsened progressively for 20 days. She underwent an upper- and lower-abdomen CT scan that revealed a heterogeneous left AI measuring 8.5 × 7 × 4.2 cm (Figure 1i). On admission, the physical examination demonstrated no abnormal findings apart from a slight increase in body temperature (37 °C). No palpable masses or skin hyperpigmentation were noted. Her past medical history was remarkable for hypertension and multiple sclerosis in a stable condition, which were treated with olmesartan, and glatiramel, respectively. Re-evaluation with CT scans demonstrated a marked increase in the dimensions of the AI (diam. 8.5 × 10 × 15 cm), with a Hounsfield scale of around 40 and a decreased wash-out period of the contrast agent. (Figure 1ii).

The laboratory assessment revealed anemia, elevated levels of lactate dehydrogenase (LDH), and slightly elevated CA-125 and β2-microglobulin, as shown in Table S1. The full hormonal evaluation is shown in Table 1. Notably, the evaluation of the adrenal function demonstrated increased serum and 24-h urinary free cortisol levels, suppressed fasting adrenocorticotropic hormone (ACTH) levels, and non-suppressed serum cortisol levels in both the overnight and the low-dose dexamethasone suppression tests, indicating autonomous cortisol secretion, as shown in Table 2. In addition, she exhibited decreased levels of serum follicle-stimulating hormone (FSH), luteinizing hormone (LH), and thyroid-stimulating hormone (TSH) for her menopausal status. Seven days after admission, the clinical status of the patient deteriorated in terms of intense night sweating, severe fatigue, and fever. CTs of the chest and pelvis and an endoscopy of upper and lower gastrointestinal systems were insignificant.

**Figure 2:**
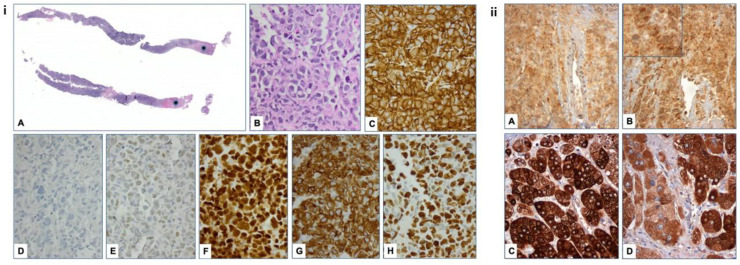
Histologic section (scanned slide; asterisks mark necrotic areas) of the fine-needle biopsy specimens from the first patient. (**i**) (**A**, H&E) of the right adrenal mass. Lymphoid tumor cells are medium to large (**Β**, H&Ε, ×400), are positive for CD20 (**C**, ×400), do not express CD10 (**D**, ×400), and are positive for BCL6 (**E**, ×400), MUM1 (**F**, ×400), and BCL2 (**G**, ×400). The Ki67 proliferation index is in the range of 80% (**H**, ×400). (**ii**) Neoplastic lymphoid cells showed CYP 17A1, mostly granular (inset in **B**), and immunopositivity (**A**,**B**, ×400). For comparison, a normal adrenal cortex (**C**, ×400) and a case of adrenocortical adenoma (**D**, ×400), both stained with the same antibody, are presented.

A CT-guided fine-needle biopsy (FNB) of the mass was performed, demonstrating areas of necrosis and a diffuse infiltrate composed of large lymphoid cells with the following immunophenotype: CD20+, PAX-5+, CD10−, BCL6+, MUM1+, BCL2+, with a Ki-67 proliferation index in the range of 80–90%. On the basis of the aforementioned findings, the diagnosis of non-germinal center B-cell–like (non-GCB) DLBCL, according to the Hans algorithm, was established (Figure 2i(A–H)).

To determine whether the lymphoma cells exhibited cortisol-secreting activity, we examined their steroidogenic profile using immunohistochemistry for the expression of 17 alpha-hydroxylase 1 (CYP17A1), as described in the Supplementary Materials and Methods; this is a key enzyme in the adrenal steroidogenic pathway, which is involved in corticoid and androgen biosynthesis. Remarkably, neoplastic cells exhibited cytoplasmic granular CYP17A1 immunoreactivity (Figure 2ii(A,B)). The normal adrenal cortex and the case of adrenocortical adenoma were stained with the same antibody and used as positive controls (Figure 2ii(C,D)). Unfortunately, due to the limited material, we were not able to analyze the protein and mRNA expression levels of other enzymes that participate in adrenal steroidogenesis.

**Figure 3:**
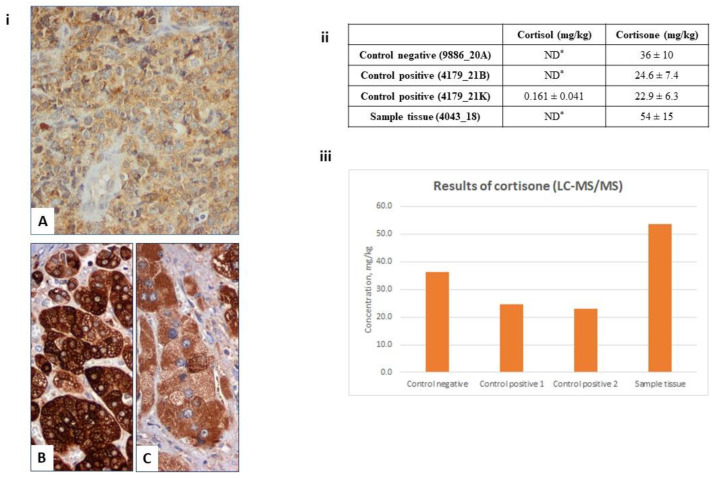
(**i**) Neoplastic lymphoid cells from the parotid gland from Case 1 showed CYP17A1, with mostly granular immunopositivity (**A**,**B**, ×400). For comparison, a normal adrenal cortex (**B**, ×400) and a case of adrenocortical adenoma (**C**, ×400), both stained with the same antibody, are presented. (**ii**,**iii**) Results from the LC-MS/MS analysis. It can be seen that cortisol was detected only in the positive control, which can explained by the origin of the tissue that secretes more cortisol compared to the other three tissues. On the other hand, cortisone was found in all of the tissues. Its presence is explained by enzymatic conversion of cortisol to cortisone. Moreover, its noteworthy that the sample from our patient presented the highest concentration of cortisone (54 mg/kg). ND*: not assessed.

Initial staging with a positron emission tomography/computed tomography (PET/CT) scan revealed an increased uptake of 18-FDG by the left adrenal (SUVmax = 19.1), as well as by a large abdominal lymphoid mass (SUVmax = 22.5). Therefore, the patient was staged as IVEA based on the Ann Arbor system and was stratified as a low-risk case according to the Revised International Prognostic Index (R-IPI) score. She was subsequently treated with immunochemotherapy with R-CHOP (rituximab, cyclophosphamide, doxorubicin, vincristine, and prednisone); the patient also received central nervous system (CNS) prophylaxis with intrathecal methotrexate. A re-evaluation of the disease by CT scans after the fourth cycle of treatment showed a small residual mass in the left adrenal (diameter 3.1 × 2.7 cm), and the hormonal evaluation showed normal levels of 24-h urinary free cortisol levels and a normal response to the overnight and low-dose dexamethasone suppression tests, as shown in Table 2. The patient completed six cycles of R-CHOP; however, the end-of-treatment PET/CT demonstrated a small residual mass in the left adrenal with high 18-FDG uptake (Deauville Score: 5). Notably, the hormonal profile of the patient at this stage was within the normal limits (Table 2). During the three following months, the patient developed a lymph node mass in the right parotid gland. A hormonal evaluation with an overnight dexamethasone suppression test and a 24-h urine test for cortisol measurement again indicated autonomous cortisol secretion. (Table 2). FNB confirmed disease progression. As was the case for the primary adrenal mass, immunohistochemical analysis for CYP17A1 revealed cytoplasmic granular CYP17A1 immunoreactivity (Figure 3(iA). Real-time quantitative PCR analysis for the detection of 11β-hydroxylase (CYP11B1), aldosterone synthase (CYP11B2), CYP17A1, and HSD3B2 mRNA transcripts (see Supplementary Materials and Methods and Table S1) that participate in normal adrenal steroidogenesis revealed no detectable expression. However, we detected cortisone using liquid chromatography tandem mass spectrometry (LC-MS/MS) (see Supplementary Materials and Methods, Table S2, Figure S1) in homogenized material from deparaffinized sections (Figure 3ii,iii).

Subsequently, the patient received three cycles of salvage immunochemotherapy with R-ESHAP (rituximab, etoposide, methylprednisolone, high-dose cytarabine, and cisplatin), achieving a complete metabolic response (CMR). This was followed by an autologous stem cell transplantation (ASCT) with carmustine, etoposide, cytarabine, and melphalan (BEAM) as a conditioning regimen. The patient remained in CMR with a normal hormonal profile five years after the ASCT.

### 2.2. Case 2

An 80-year-old female presented with lumbar pain for three months, which was initially alleviated with analgesic medications; however, due to increased pain intensity, she underwent CT imaging of the upper and lower abdomen, which demonstrated a bilateral AI (right: 9 × 6.5 cm, left: 3.6 × 3.2 cm) (Figure S1i). In the meantime, the patient’s clinical status deteriorated, as she exhibited symptoms of vomiting, fatigue, weakness, and abdominal pain, which led her to be brought to our hospital for further investigation and treatment. Her past medical history was remarkable for instances of chronic atrial fibrillation, which was treated with acenocoumarol and metoprolol.

On admission, the physical examination showed significant results in terms of the decreased breath sounds of the lower right lobe of the lung, proximal muscle weakness, and cardiac arrhythmia. No palpable mass or skin hyperpigmentation were noted.

The laboratory assessment showed no pathological findings, as shown in Table S1. The hormonal evaluation showed increased serum and 24 h urinary free cortisol values, suppressed ACTH levels, and non-suppressed serum cortisol levels in both the overnight and the low-dose dexamethasone suppression tests, indicating autonomous cortisol secretion, as shown in Table 2.

The chest CT illustrated moderate right pleural effusion; there was no evidence of a lung tumor or mediastinal lymph node enlargement, while the brain CT scan was insignificant. A second CT scan of the abdomen after 20 days revealed a rapid progression of both masses (right: 15 × 10 × 11 cm, left: 4 × 4.5 × 6 cm), with mass effects on the surrounding tissues and lymph node enlargement around the masses, with a Hounsfield scale of around 35, and a decreased washout period of the contrast agent (Figure S1ii).

During her hospital stay, the patient exhibited further clinical deterioration, in parallel with a progressive increase in cortisol production. An ultrasound-guided FNB of the AI established the diagnosis of non-GCB DLBCL (Figure S1iii(A–I)). The lymphoid tumor cells were medium to large in size, mostly with centroblastic cytomorphology, with the following immunophenotype: CD20+, PAX-5+, CD10−, BCL6+, MUM1+, BCL2+, MYC−. Meanwhile, the proliferation Ki67 index was high, in the range of 80%. Unfortunately, after the diagnostic antibody panel, no material was available for further stains, e.g., with the anti-CYP17A1 antibody. The patient was staged as IVEA based on the Ann Arbor system, and she was stratified as low-risk according to R-IPI score. She was subsequently treated according to an R-CNOP regimen (rituximab, cyclophosphamide, mitoxantrone, vincristine, prednisone); however, shortly thereafter, she developed febrile neutropenia and died from septicemia attributed to multi-resistant Klebsiella *pneumoniae*.

## 3. Discussion

In this report, we described two cases of PAL presenting as AI with autonomous cortisol secretion, which fluctuated in parallel with the disease course. Notably, in the first case, we demonstrated that cortisol secretion was derived from neoplastic cells at both the point of diagnosis and relapse.

PAL has mainly been associated with adrenal insufficiency (50–69%) due to mass effects on the adrenals, and the primary presenting symptoms include hypotension, hyponatremia, fatigue, skin hyperpigmentation, and vomiting [3]. Remarkably, our cases showed autonomous cortisol production, evidenced by increased basal serum and 24-h free urinary cortisol levels, suppressed basal ACTH levels, and non-suppressed cortisol levels in both the overnight and the low-dose dexamethasone suppression tests. Additionally, in the first case, the neoplastic lymphoid cells showed evidence of the expression of the steroidogenic enzyme CYP17A1, both at diagnosis and upon progression. Moreover, cortisone was detected using LC-MS/MS, further supporting autonomous cortisol production by the lymphoma cells. Furthermore, the first patient showed lower serum FSH, LH, and TSH levels for her menopausal status at diagnosis; these levels increased after immunochemotherapy, indicating that cortisol overproduction had an effect on these levels. The best evidence of autonomous cortisol production related to DLBCL is the normalization of the findings after control of the disease with immunochemotherapy in the first case, which became abnormal upon relapse and again normalized in the second CR.

To our knowledge, this is the first report of steroidogenesis in neoplastic lymphoid cells. Under physiological conditions, steroidogenesis is metabolically active not only in the adrenal glands but also in extra-adrenal tissues such as in the Leydig cells of the testis, in the granulosa and theca cells of the ovaries, and in the syncytiotrophoblast cells of the placenta [10]. Furthermore, other tissues and organs, such as the CNS [11], skin [12], lungs [13], intestine [14,15], and thymus [16,17], have been shown to produce steroids, and this local production seems to represent an immunoregulatory mechanism for controlling the harmful effects of an excessive activation of the immune system [18,19].

In addition to normal tissues, the activation of steroidogenesis has been described in colon cancer cells as an immunomodulatory mechanism of immune escape and cancer progression [20]. Similarly, the adrenal gland offers an immunosuppressive microenvironment for metastatic microsatellite high-instability colorectal cancer, acting as a sanctuary site for immunotherapy resistance [21]. Regarding adaptive immune cells, although induced steroidogenesis has been reported in T lymphocytes under infectious [22] and neoplastic conditions [23], steroid production by neoplastic lymphoid cells has not been reported previously.

Cortisol synthesis and release by DLBCL cells might represent a novel mechanism of immune evasion. B lymphocytes are professional antigen-presenting cells and, during their neoplastic transformation and progression, additional adaptive immune resistance mechanisms might be activated by regulating T-cell function in order to limit anti-tumor immune responses. Additional studies are needed to analyze steroidogenesis in B-cell lymphomas, which might only locally modify the immune microenvironment without clinically detectable Cushing’s syndrome.

In conclusion, PAL can present as Ais, which are most often bilateral; although rare, these entities should be considered in the differential diagnosis of AI, especially in cases with rapidly growing masses and nonspecific symptoms such as fatigue, weakness, and night sweats. Hormonal evaluations should be performed in all cases of PAL. Histopathology is the only effective method for PAL diagnosis. Investigations of larger series of primary adrenal and extra-adrenal DLBCL will shed light on the incidence of steroidogenesis by neoplastic B-cells and the possible presentation of DLBCL cases as subclinical Cushing’s syndrome. Furthermore, analyses of the immune microenvironment in these cases will shed light on the possible role of steroidogenesis as an adaptive immune resistance mechanism, which could then be explored for new therapeutic interventions.

## Figures and Tables

**Table 1:** Hormonal evaluation of the patients at baseline and following treatment.

Hormone	Case 1		Case 2
Time	At Diagnosis	After 6 Cycles of R-CHOP	At Diagnosis
Total serum renin (μIU/mL, n.v. 3–40)	6	10	288
Plasma aldosterone (ng/dL, n.v. 3–20)	9	14	10
Urinary VMA (mg/24 h, n.v. <8)	4.6	4	7.6
Urinary free metanephrines (μg/24 h, n.v. 20–350)	56	48	75
Urinary normetanephrines (μg/24 h, n.v. 30–600)	303	187	519
17 (OH)Progesterone (ng/mL, n.v. 0.5–1.2)	0.6	0.8	ND
Testosterone (ng/mL, n.v. 0.029–0.408)	0.16	0.13	0.25
Dehydroepiandrosterone sulfate (μg/dL, n.v. 52–252)	96	56.9	10.9
FSH (mIU/mL, n.v. 2.5–12.5)	24.7	66.7	22.93
LH (mIU/mL, n.v. 3.5–13.5)	15.1	42.9	6.5
Estradiol (pg/mL, n.v. <5)	16	<5	19.6
TSH (μIU/mL, n.v. 0.274.2)	1.03	3.7	1.26

ND: not assessed.

**Table 2:** Evaluation of adrenal function of the cases at diagnosis and at various timepoints during and after treatment.

	Serum Cortisol after Overnight Dexamethasone Suppression Test (1 mg Dexamethasone) (n.v. <1.8 μg/dL)	Serum Cortisol at Midnight (μg/dL, n.v. <7.2)	Urinary Free Cortisol (μg/24 h, n.v. <50)	Plasma ACTH (pg/mL, n.v.: 5–60 pg/mL)	Serum Cortisol (μg/dL, n.v. 6.2–19.4)
Case 1					
At diagnosis	28	22	179	5	20.3
In CRu after 4 cycles of R-CHOP	1.9	4.1	55	50	21.4
In CRu, after 6 cycles of R-CHOP	2	4	62	50	26.1
At relapse	10	15	252	6	30
In CMR, after auto-SCT	1.3	3.8	42	69	19.4
Case 2					
At diagnosis	4	24	361	19.8	21.6
After 1 cycle with R-CHOP	ND	ND	ND	63	>63

ND: not assessed.

## Data Availability

No new data were created or analyzed in this study. Data sharing is not applicable to this article.

## Supplementary Materials

The following supporting information can be downloaded at: https://www.mdpi.com/article/10.3390/jcm12155032/s1, Supplementary Materials [24]; Table S1. Clinical and laboratory data of the 2 cases at diagnosis; Table S2. Primers used for RT-PCR for the detection of CYP11B1, CYP11B2, CYP17A1 AND HSD3B2, mRNA transcripts that participate in normal adrenal steroidogenesis; Figure S1. CT scan without contrast and histologic section (scanned slide) of the fine needle biopsy specimens from the second patient (i) A large mass sized 9 × 6.5 cm on the right adrenal and another mass sized 3.6 × 3.2 cm on the left adrenal gland on admission. (ii) Progression of both masses size (Right adrenal mass 15 × 10 × 11 cm and Left adrenal mass 4 × 4.5 × 6 cm). (iii) (A, H & E) of the left adrenal mass from case 2. Lymphoid tumour cells are medium to large (Β, H & E, × 400), are positive for CD20 (C, × 400), does not express CD10 (D, × 400) and are positive for BCL6 (E, × 400), MUM1 (F, × 400) and BCL2 (G, × 400), whereas about 30% of tumour cells are positive for MYC (H, × 400). The Ki67 proliferation index is in the range of 80% (I, × 400).
