# Supplementary material for: Primary Adrenal Lymphomas with Cushing’s Syndrome: Two Cases with Evidence of Endogeneous Cortisol Production by the Neoplastic Lymphoid Cells
Source: J Clin Med. 2023 Jul 31;12(15):5032. doi: 10.3390/jcm12155032 (PMC10419581; doi:10.3390/jcm12155032)
Supplement: Supplementary file 1 [file jcm-12-05032-s001.zip › jcm-2487219-SI.pdf]

Journal of
Clinical Medicine

MDPI

Supplementary Materials: Primary Adrenal Lymphomas with Cushing's Syndrome: Two Cases with Evidence of Endogeneous Cortisol Production by the Neoplastic Lymphoid Cells

Sotirios G. Papageorgiou, Ioanna Mavroeidi, Marios Kostakis, Aris Spathis, Danai Leventakou, Evangelia Kritikou, Nikolaos Oikonomopoulos, Chrysoula Kourkouti, Maria Krania, Anthi Bouchla, Thomas Thomopoulos, Zoi Tsakiraki, Konstantinos Markakis, Ioannis G. Panayiotides, Nikolaos Thomaidis, Vasiliki Pappa, Periklis G. Foukas and Melpomeni Peppa

Supplementary Materials and Methods

Immunohistochemical analysis for CYP17A1

Immunostaining was performed on 3μm paraffin sections. Primary antibody against CYP17A1 (Proteintech, rabbit polyclonal antibody, catalog number: 14447-1-AP, dilution 1:100), was used. For deparaffinization, rehydration and antigen retrieval, slides were immersed in a low pH target retrieval solution (Envision Flex, K8005, DAKO) in the PT Link module, DAKO, for 20 minutes and cooled at room temperature for 20 minutes. Subsequently, the slides were placed in the DAKO Link-48 autostainer and the following incubation steps were performed: incubation with anti-CYP17A1 primary antibody for 30 min, enhancement with Envision Flex+ Rabbit (DAKO, Linker SM805) for 15 min, endogenous peroxidase activity blocking reagent, peroxidase conjugated Envision flex/HRP [K5007, DAKO], EnVision Flex substrate buffer (code no. SM803) and EnVision Flex DAB+ chromogen (code no. DM827). All sections were counterstained with hematoxylin for 45 seconds prior to mounting.

Real-time quantitative PCR analysis for 11β-hydroxylase (CYP11B1), aldosterone synthase (CYP11B2), CYP17A1 and HSD3B2 mRNA expression.

Real-time quantitative PCR was conducted to analyze the expression of the 11β-hydroxylase (CYP11B1), aldosterone synthase (CYP11B2), CYP17A1 and HSD3B2 genes in deparaffinized sections from lymph node mass of parotid gland of first patient and normal adrenal gland as positive control. Briefly, total RNA was extracted with AllPrep DNA/RNA FFPE Kit(QIAGEN). cDNA was synthesized from 5 mg total RNA using the SuperScript First-Strand (Invitrogen). PCR amplification assays were performed with the PowerUp™ SYBR™ Green Master Mix (applied biosystems on an Rotor-Gene Q Real-Time PCR System (Applied Bio-systems). Relative quantification was determined by normalizing the expression for each gene to β-actin gene. The primers used shown in suppl. Table S2.

Liquid chromatography tandem mass spectrometry LC-MS/MS

Sample preparation

The samples were stored at 4 °C before analysis. For the extraction and isolation of cortisol and cortisone from the tissue samples, a protocol proposed by Rönquist-Nii et al. (1) was applied with some modifications. Briefly, the sample was weighed into a 15 mL centrifuge tube and kept for 30 min at – 80 °C. Afterwards, 5 mL ethyl acetate (Fisher Chemical, UK) was added in the frozen tissue, the mixture was shaken for 15 min by a rotator drive model STR4 (Stuart, UK) and then centrifuged at 4000 rpm and 4 °C for 10 min. The upper phase was transferred to a glass tube and evaporated to dryness under nitrogen gas at 40 °C. 1 mL of heptane (Carlo Erba, France) was added and the tube was vortexed. Also, 1 mL of methanol:water (7:2, v/v) mixture was added and the tube was vortexed again. The upper phase (heptane) was discarded, and the lower phase was evaporated to 0.2 mL under nitrogen gas at 40 °C. The solution was injected into the chromatographic system. Three measurements ($n = 3$) were performed for each sample.

Instrumentation

Cortisol and cortisone were determined with liquid chromatography tandem mass spectrometry system (LC-MS/MS). The LC-MS/MS system consisted of AB SCIEX Exion AD Series System connected with AB SCIEX QTRAP 5500+ hybrid linear ion-trap triple quadrupole mass spectrometer equipped with a Turbo V Spray source (Toronto, Canada). Separation was performed using an Atlantis T3 column (3 μm, 2.1 mm × 100 mm) from Waters (Milford, MA, USA). The injection volume was 10 μL and oven temperature was maintained at 30 °C. The mobile phase consisted of 5 mM ammonium acetate and methanol (25:75, v/v) and was delivered isocratic with a flow rate of 0.25 mL/min. Mass spectrometry conditions were as follows: curtain gas (CUR) 20 psi, collision gas (CAD) 6 psi, ionspray 5500 V, temperature 500 °C, ion source gas 1 (GS1) 60 psi, ion source gas 2 (GS2) 45 psi and dwell time was 50 msec. The following MRM transitions in a positive mode were selected: m/z 363.2→121.1 (cortisol 1), 363.2→327 (cortisol 2) and m/z 361.2→332.4 (cortisone 1), 361.2→343.2 (cortisone 2). The declustering potential (DP), entrance potential (EP), collision energy (CE) and collision cell exit potential (CXP) were optimized at 79 V, 10 V, 33 V and 14 V for cortisol 1, 79 V, 10 V, 23 V and 26 V for cortisol 2, 111 V, 10 V, 11 V and 12 V for cortisone 1, 111 V, 10 V, 15 V and 26 V for cortisone 2. Ionspray voltage (IS) and temperature were set at 5500 V and 500 °C, respectively. Peak areas of each analyte were obtained using Analyst 1.7.2 data processing software (AB SCIEX, Toronto, Canada), while the concentrations of compounds were calculated using a matrix matched sample.

References

1. Ronquist-Nii, Y.; Edlund, P.O. Determination of corticosteroids in tissue samples by liquid chromatography–tandem mass spectrometry. *J. Pharm. Biomed. Anal*. **2005**, *37*, 341–350.

Table S1. Primers used for RT-PCR for the detection of CYP11B1, CYP11B2, CYP17A1 AND HSD3B2, mRNA transcripts that participate in normal adrenal steroidogenesis.

CYP11B1	5′-AATGCGGAACTGTCGCCAGATG-3′ (forward)
	5′-TCAGCAAGGGAAACACCGTC-3′ (reverse)
CYP11B2	5′-ACTCGCTGGGTCGCAATG-3′ (forward)
	5′-AGTGTCTCCACCAGGAAGTGC-3′ (reverse)
CYP17A1	5′-CCACCTTTGCCCTGTTCAAG-3′ (forward)
	5′-GCCAGCATATCACACAATGTACTG-3′ (reverse)
HSD3B2	5′-AGGACCAAGCTGACTGTACTT-3′ (forward)
	5′-TAGATGA AGACTGGCACACTGG-3′ (reverse)
β-actin	5′-TCCCTGGAGAAGAGCTACG-3′ (forward)
	5′-GTAGTTTCGTGGATGCCACA-3′ (reverse)

Table S2. Clinical and laboratory data of the 2 cases at diagnosis.

**Variable**	**Case 1**	**Case 2**
Age (yrs)	52	80
Gender	Female	Female
BMI (kg/cm²)	22.1	27
Blood Pressure (mmHg)	120/70	110/70
Hemoglobin (g/dL, n.v. 12–15)	9.6	13.7
WBC (g/dL, n.v. 4000–11,000)	6840	6290
NEUT/LYMPH/MONO/EOS (%)	68/17/11/2	62/18/17.8/1.3
PLT (K/μL, n.v. 150–400)	390	251
Glucose (md/dL, n.v. 74–106)	98	96
Urea (mg/dL, n.v. 16.6–48.5)	16.3	48.6
Creatinine (mg/dL, n.v. 0.5–0.9)	0.6	0.9
Billirubin (mg/dL, < 1.2)	0.23	0.49
γ-GT (U/L, n.v. 5–36)	22	36
Alkaline Phospatase (U/L, n.v. 35–104)	51	51
LDH (U/L, n.v. 135–225 U/L)	386	708
Na (mmol/n.v. 136–146 mmol/L)	141	134
K (mmol/L, n.v. 3.5–5.1)	4.5	4.7
Ca (mg/dL, n.v. 8.6–10.3)	9.5	N/A
INR (n.v 0.9–1.6)	1.1	N/A
C-Reactive Protein (mg/L, n.v. 0–6)	25.1	3.17
Aspartate transaminase (U/L, n.v. 0–32)	18	25.1
Alanine transaminase (U/L, n.v. 0–33)	14	65/22
Carcinoembryonic antigen (ng/mL, n.v. < 3.8)	0.8	1.1
Cancer antigen 125 (U/mL, n.v. < 35)	55.9	20.5
Alpha-fetoprotein (ng/mL, n.v < 7)	4.6	2.5
Cancer antigen 19–9 (U/mL, n.v. < 27)	<0.6	<0.6
β2- microglobulin (mg/L, n.v. 1–2.5)	3	N/A

N/A = not assessed.

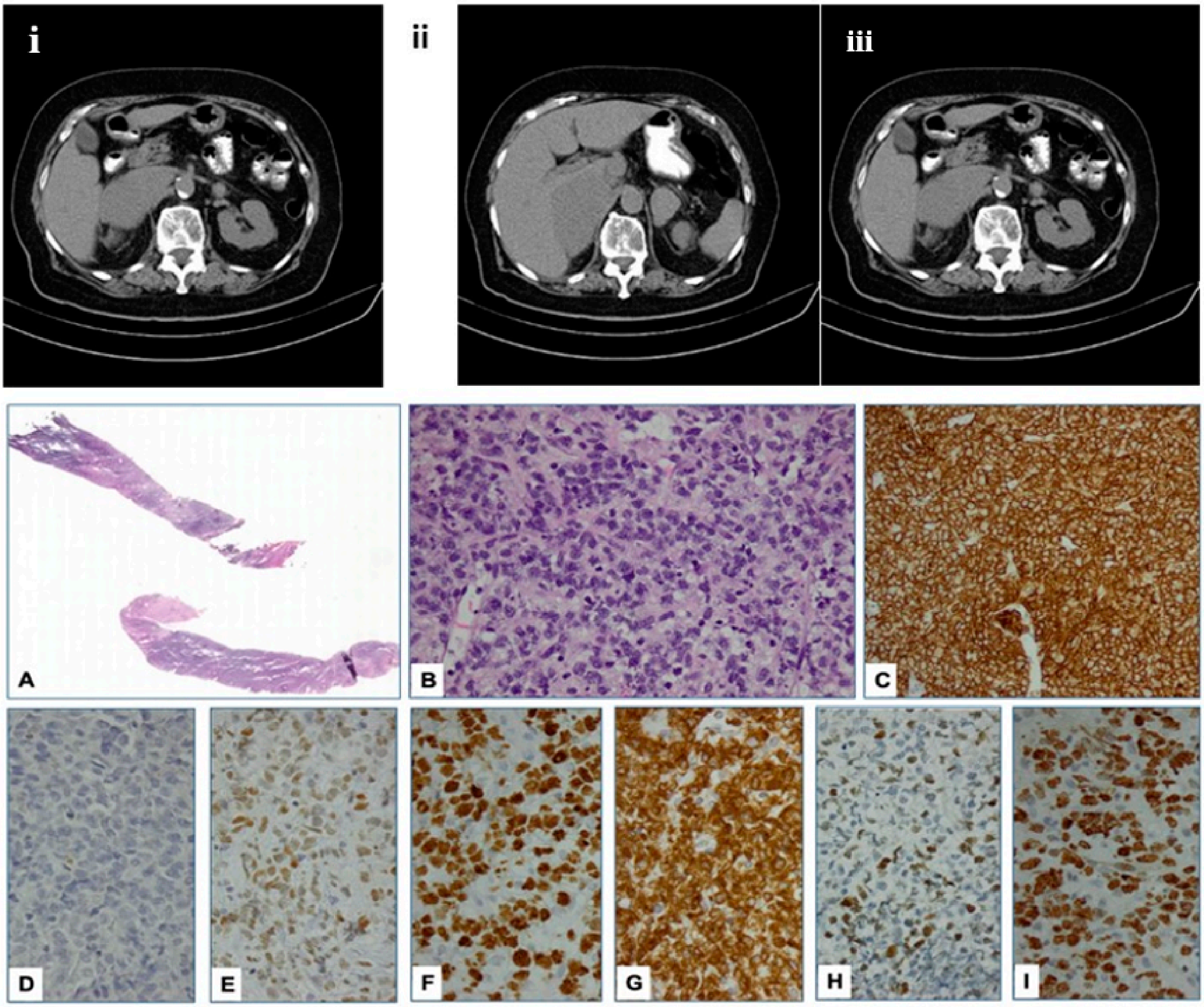

Figure S1. CT scan without contrast and histologic section (scanned slide) of the fine needle biopsy specimens from the second patient (**i**) A large mass sized 9 × 6.5 cm on the right adrenal and another mass sized 3.6 × 3.2 cm on the left adrenal gland on admission. (**ii**) Progression of both masses size (Right adrenal mass 15 × 10 × 11 cm and Left adrenal mass 4 × 4.5 × 6 cm). (**iii**) (**A**, H & E) of the left adrenal mass from case 2. Lymphoid tumor cells are medium to large (**B**, H & E, × 400), are positive for CD20 (**C**, × 400), does not express CD10 (**D**, × 400) and are positive for BCL6 (**E**, × 400), MUM1 (**F**, × 400) and BCL2 (**G**, × 400), whereas about 30% of tumor cells are positive for MYC (**H**, × 400). The Ki67 proliferation index is in the range of 80% (**I**, × 400).
